# Comment on “Urinary NGAL as a Diagnostic and Prognostic Marker for Acute Kidney Injury in Cirrhosis”

**DOI:** 10.14309/ctg.0000000000000382

**Published:** 2021-07-12

**Authors:** Fangbin Weng

**Affiliations:** 1 Department of Infectious Diseases, YiWu Central Hospital, Zhejiang, China.

We read with interest the study by Allegretti et al. recently published in *Clinical and Translational Gastroenterology* regarding the use of urinary neutrophil gelatinase-associated lipocalin (NGAL) in discerning the phenotypes of acute kidney injury (AKI) and predicting the short-term outcome in patients with decompensated cirrhosis (1). This prospective study demonstrated that urinary NGAL can differentiate acute tubular necrosis (ATN)-AKI from prerenal and hepatorenal syndrome (HRS)-AKI at an optimal cut point. And, it was also shown that urinary NGAL performed better in predicting 90-day transplant-free survival than the model for end-stage liver disease (MELD) score. The study provided evidence for the clinical utility of urinary NGAL as a diagnostic and prognostic marker for AKI in cirrhosis. But, the following points need clarification.

The major concern is the difficulty to make a differential diagnosis among prerenal AKI, ATN-AKI, and HRS-AKI in patients with decompensated cirrhosis. For example, infection is a common precipitating event causing HRS and was present in 19.3% patients with prerenal AKI in the study (1). As infection must be treated, it is not clear in at least some of these patients that the resolution of AKI was attributed to volume administration or the resolution of infections. To be more complicated, when treating infections, some antibiotics, such as fluoroquinolones and cephalosporins, are potentially nephrotoxic and may cause ATN-AKI (2). Thus, in these cases, an exclusive diagnosis of prerenal AKI can be challenged. Actually, in an *a priori* study investigating the cause of renal failure in patients with decompensated cirrhosis, 17.6% of patients could not be classified into a single group because of the presence of a combination of several causes or miscellaneous conditions (3). Second, it is impressive that NGAL alone had better discriminative performance in predicting 90-day transplant-free mortality than the MELD, MELD-Na, and CLIF-C ACLF score. However, it should be stated that the studied population mainly consisted of patients with AKI (161/213, 75.6%) and therefore was divergent from general population of decompensated cirrhosis. Finally, it is not clear that how the reference group was matched with the AKI group, which may introduce selection bias.

## CONFLICTS OF INTEREST

**Guarantor of the article:** Fangbin Weng, MD.

**Specific author contributions:** F.W.: conceived the idea and wrote the article.

**Financial support:** None to report.

**Potential competing interests:** None to report.
